# What Are the Thoughts of the Dying?

**Published:** 1889-08

**Authors:** 


### WHAT ARE THE THOUGHTS OF THE DYING?

In the Société de Biologie Féré affirmed that a dying person in his last moments thinks of the chief events of his life. Persons resuscitated from drowning, epileptics with grave attacks, persons dying and already unconscious, but momentarily brought back to consciousness by ether injections to utter their last thoughts, all acknowledge that their last thoughts revert to momentous events of their life. Such an ether injections revives once more the normal disposition of cerebral activity, already nearly extinguished, and it might be possible at this moment to learn of certain important events of the past life. Brown-Sequard mention the remarkable fact that persons who, in consequence of grave cerebral affections have been paralyzed for years, get back at once when dying their sensibility, mobility, and intelligence. All such facts clearly show that at the moment of dissolution, important changes take place, reacting upon the composition of the blood and the functions of the organs.—*Wien. Med. Zeitung.*

